# Effectiveness of Virtual Reality and Interactive Simulators on Dental Education Outcomes: Systematic Review

**DOI:** 10.1055/s-0041-1731837

**Published:** 2021-08-24

**Authors:** Rania Moussa, Amira Alghazaly, Nebras Althagafi, Rawah Eshky, Sary Borzangy

**Affiliations:** 1 Department of Substitutive Dental Sciences, College of Dentistry, Taibah University, Medinah, Saudi Arabia; 2 Department of Restorative Dental Sciences, College of Dentistry, Taibah University, Medinah, Saudi Arabia; 3 Department of Pediatric Dentistry and Orthodontics, College of Dentistry, Taibah University, Medinah, Saudi Arabia

**Keywords:** augmented reality, simulation, dental education, haptics, students, dental, virtual reality, education

## Abstract

In recent years, virtual reality and interactive digital simulations have been used in dental education to train dental students before interacting with real patients. Scientific evidence presented the application of virtual technology in dental education and some recent publications suggested that virtual and haptic technologies may have positive effects on dental education outcomes. The aim of this systematic review was to determine whether virtual technologies have positive effects on dental education outcomes and to explore the attitudes of dental students and educators toward these technologies. A thorough search was conducted in PubMed, Scopus, MEDLINE (via EBSCO), The Cochrane Library (via Wiley), Web of Science Core Collection (via Thomson Reuters), and Dentistry and Oral Science source (via EBSCO) using the keywords (student, dental) AND (education, dental) AND (virtual reality) OR (augmented reality) OR (haptics) OR (simulation) AND (dentistry) OR (dental medicine). The quality of the reported information was assessed following the Preferred Reporting Items for Systematic Review and Meta-Analysis (PRISMA) statement for systematic reviews. A total of 73 publications were considered for this review. Fifty-two of the selected studies showed significant improvement in educational outcomes and virtual technologies were positively perceived by all the participants. Within the limitations of this review, virtual technology appears to improve education outcomes in dental students. Further studies with larger samples and longer term clinical trials are needed to substantiate this potential positive impact of various virtual technologies on dental education outcomes.

## Introduction

In recent years, virtual reality (VR) simulations have been employed in dental education as an adjunctive to the traditional skill training curriculum to train dental students before interacting with actual patients. 1 2 Dental education differs from any other form of medical education as it is a combination of theory, laboratory, and clinical practice. The challenge in dental education arises from the fact that theoretical knowledge acquisition requires spatial imagination and the patient-centered training on traditional mannequin simulation does not resemble realistic clinical situations. 3 Preclinical and clinical training is of paramount importance for developing fine motor skills to prepare dental students to engage in the dental profession. Many of the required dental education competency skills are challenging to acquire, and mandates repeated training and long practice. 4 Since the breakthrough of the novel coronavirus SARS-Co-V-2 (severe acute respiratory syndrome coronavirus 2) in late 2019, 5 all essential activities were affected, calling for social distancing, and the traditional dental teaching models of one-on-one pedagogical design had to be partially replaced by digital or virtual setups to avoid the gathering of the youth in closed spaces.

VR is gaining acknowledgment as a valuable tool for training dental students, and its use by dental schools is rising worldwide. 6 VR is defined as a computer-generated medical simulation of a three-dimensional (3D) image or environment that uses software to create an immersive computer-generated environment. Users put on a head-mounted display that places them inside an experience, where they can engage with the setting and virtual characters in a way that feels real. VR could be beneficial in dental education, permitting a patient noncontact training environment. 1 2

Augmented reality (AR) is a superimposition of computer-generated graphics over a real-life scene. It differs from VR, which does not demonstrate natural conditions. AR refers to a form of technology that integrates both real and virtual elements in a combined experience and allows learners to visualize complex spatial relationships, abstract concepts, and experience phenomena that might have been impossible in the real world, especially in surgical procedures coaching. 7 8 Immersive virtual reality (IVR) is one form of AR where the user interacts with a digital 3D environment recreated through 360 degrees actual records. 9

Haptic technology (HT) is a more recent simulation that involves tactile sensation while interacting with computer-generated objects. Haptics means the sense of touch and consists of the science of incorporating the interaction with the external environment through contact. 2 Implementing these technologies in dental education motivated designers to create virtual teeth with and without pathology, multilayered and featured with different mechanical hardness for enhanced reality. 10 11

The applications of VR in dental education attracted the attention of researchers even in the early experimental stages. 7 It was suggested that it could enhance dental education compared with traditional teaching, 1 especially in the training of restorative dentistry, 12 13 and dental surgery, 14 15 although it may expand to include endodontics and orthodontics. 16 17 18 VR enabled the delivery of distant online lectures through 3D VR workplace. The flexibility of the technology allowed the attendees’ active contribution and facilitated 3D understanding of surgery and related anatomy, despite the limitation of technical issues. 19 However, the results of VR effectiveness in dental education outcomes are controversial. Thus, this systematic review aimed to evaluate the effectiveness of VR simulations on dental education outcomes. The assessed results of VR interventions were knowledge, clinical skills, attitude, and satisfaction of both learners and educators.

## Methods

### Protocol and Eligibility Criteria

This systematic review was conducted according to the Preferred Reporting Items for Systematic Review and Meta-Analysis (PRISMA) guidelines. 20 A modified PICOS search was defined, and studies that fulfilled the following criteria were selected:

1. Population (P): Undergraduate and postgraduate dental learners enrolled in any dental-related education or training program were included in the review.
2. Intervention (I): Virtual simulation teaching and assessment methods including but not limited to VR, AR, and HT.
3. Primary outcomes (O): Include clinical competencies measured pre or post intervention represented in learners’ knowledge and manual skills. Secondary outcomes included students’ and educators’ perceptions of VR designs.
4. Study design (S): the review applied no limits for the study design.
5. Comparison (C): was not a mandatory item to include a study in this review.

### Information Sources

A systematic electronic search was performed limited to English language articles published between January 2010 to the end of March 2021. Studies were identified by searching the following electronic databases for relevant studies: PubMed, Scopus, MEDLINE (via EBSCO), The Cochrane Library (via Wiley), Web of Science Core Collection (via Thomson Reuters), and Dentistry and Oral Science source (via EBSCO).

The following search terms were used for identification of eligible studies: (student, dental) AND (education, dental) AND (VR) OR (AR) OR (haptics) OR (simulation) AND (dentistry) OR (dental medicine). Keywords were adjusted for use with each of the databases mentioned earlier. Further electronic search of the relevant articles in the Journal of Dental Education and the European Journal of Dental Education was performed while running our electronic search. The bibliographies of the revealed full texts, were manually searched for additonal studies.

### Study Selection

The search results were combined in a single Mendeley library (Mendeley Desktop v1.19.6) and duplicates were excluded. Two authors independently screened titles, abstracts to identify potentially eligible studies. Exclusion criteria included preliminary reports, reports without an underlying study design, and studies describing the software or hardware of the virtual technology. One co-author retrieved full-text versions of the selected studies. Selected publications were independently reviewed by two investigators.

### Data Collection

Customized forms following the guidelines of the Cochrane Consumers and Communication Review Group template for review authors, 21 were used to record the following data from the selected studies:

- Characteristics of the study: study design, research country, and time of intervention (before-after).
- Characteristics of the study participants: number of participants, stage of education (under or postgraduate), and year of study.
- Virtual intervention applied: dental specialty where simulation was used, type of the system, and the source of virtual simulations: whether access to virtual simulation was from home or at academic laboratories.
- The outcome investigated; subjective or objective assessment, and the tools used to measure the output.
- Results of the selected studies.

## Results

### Studies Included

**Fig. 1:**
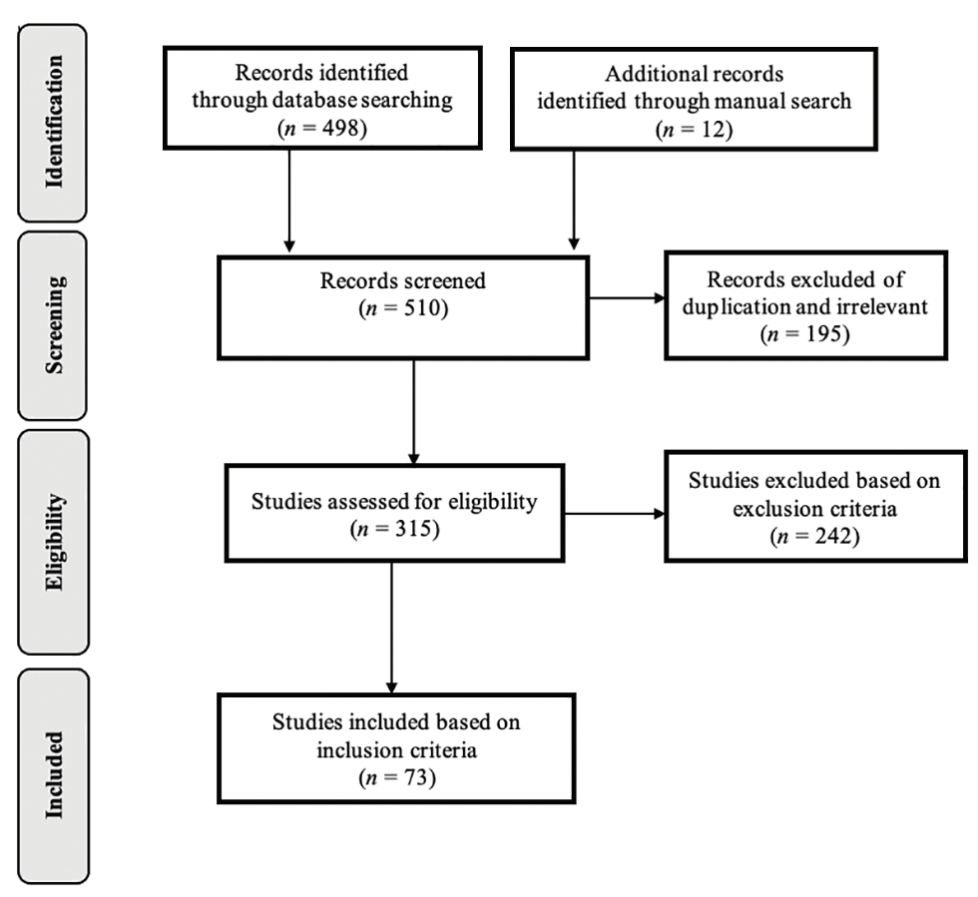
Preferred Reporting Items for Systematic Reviews and Meta-Analyses (PRISMA) diagram flow of the selection process.

The study selection process for inclusion in this review is summarized in Fig. 1 (diagram flow). The database search strategy identified 498 potentially eligible references. Twelve additional articles were included after review of references. Duplicates were excluded. After screening titles, abstracts, 437 articles were excluded applying the exclusion criteria. Eventually 73 studies were included in the review that included 5,275 participants.

**Fig. 2:**
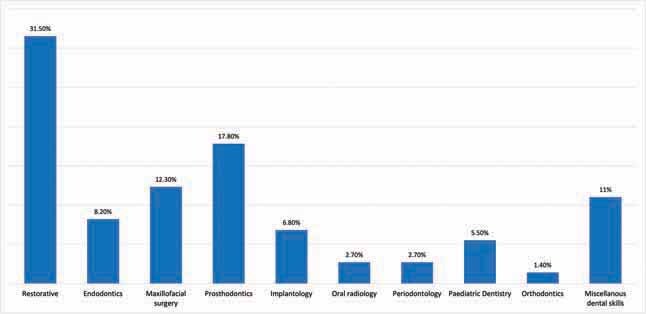
Bar chart percentile representation of each dental specialty in the selected studies.

The retrieved studies were categorized according to the field of dental education in which VR was applied. Fig. 2 shows the percentile representation of each dental specialty in the selected studies.

### Description of the Study Characteristics

#### Restorative Dentistry

Twenty-three of the selected studies applied VR in restorative dentistry with total included participants, *n* = 2,201, in which 62.1%, *n* = 1,367 were first year dental students. The detailed characteristics of the included studies are shown in Table 1 . HT was the most used in 18 of the selected studies, 12 22 23 24 25 26 27 28 29 30 31 32 33 34 35 36 37 38 VR simulator in three studies 39 40 41 and AR, 13 and interactive video games, 42 one study each. Access to all these technologies was through academic laboratories except in one study. 13 In the selected studies, students’ manual skills was the most common tested outcome represented in cavity preparations in 52.17%, *n* = 12, 13 24 25 28 29 30 33 34 35 38 39 41 or geometric figures 34.78%, *n* = 8. 12 22 23 26 27 31 32 36 Other manual skills tested were dentin etching and resin bonding, 42 and zinc phosphate cement application, 40 one study each. Four studies assessed VR on theoretical knowledge. 13 37 40 42 Results showed significant difference in 14 of the selected studies in manual clinical skills 12 13 23 27 29 30 31 34 35 36 38 39 40 41 and two studies in theoretical knowledge. 37 40

**Table 1:** Characteristics of the selected studies in restorative dentistry

S. no	Author, Year, Country	VR system	Participants	Study design	Tool of assessment	Tested outcome	Results
1	Urbankova 2010, UK 39	Adjunctive computerized dental simulator (CDS)	(75) 1st year DS	RCT	Class I and II cavity preparation	Timing on exam performance	CDS significantly better than controls on exams 1 and 2 but not significant on exam 3
2	Urbankova and Engebretson 2011, UK 22	Haptic simulator	(39) 1st year DS	CS	Perceptual ability test (PAT) Geometric figures haptic exercises	Accuracy, time, and success rate	Correlation is nonsignificant between PAT and exam scores, and significant between exam scores, time and accuracy
3	Amer et al 2011, United States 42	Interactive dental video game to teach dentin bonding	(80) 1st year DS	RCT	Pre and post written examination Shear bond strength test Students’ perception	Knowledge and clinical skills	No significant difference in knowledge or clinical skills except in wetness of dentine following etching. Students accredited the method of teaching
4	Urbankova et al 2013, UK 31	Complex haptic Simulator	(39) 1st year DS	CST	Haptic exercise of geometric figures Plastic tooth preparations	Accuracy and time Quality of plastic-tooth preparation	Number of failures in haptic exercises showed significant predictor of examination scores
5	Bakr et al 2014, Australia 32	Simodont haptic (3D-VR)	(42) 2nd year DS	CCO Early or late haptic training	pre- and post-psychomotor skills test Pre- and post-experimental and flow questionnaires Class II amalgam preparation on permanent 1st molar	% of target area prepared. Expectations, and attitudes. Quality of prepared cavity	No significant difference in practical test (pre and post) between groups. The system was highly accepted by the students
6	Koo et al 2015, United States 33	Haptic device (SensAble)	(34) 2nd year DS	RCT	Class II amalgam and class III resin Questionnaire	Cavity outline and integrity of adjacent tooth. Subjective evaluation of the simulation	Non-statistically significant post haptic scores. Game-feature of the device made the learning experience more interesting
7	Cox et al 2016, UK 34	HapTEL system	(101) 1st year DS	CS	Virtual caries lesions with increased complexity	% of caries removed, healthy tissue remaining, pulp exposure, and drilling time	% caries tissue removed, healthy tissue remaining, and pulp exposure improved for over 90%
8	San Diego et al 2016, UK 35	HapTEL system	(120) 1st year DS	CST	Carries removal tasks with increasing complexity	% of caries removed; healthy tissue remaining; pulp exposure, drilling time	Significant increase in % of carries removed, less pulp exposure, and less preparation time
9	de Boer et al 2016, Netherlands 36	Simodont Haptic dental trainer	(124) 1st year DS	CCO	Cross-figure preparation Manual dexterity exercise with 2D or 3D vision Questionnaire	Rate of success	3D vision achieved significantly better results than 2D. Over 90% preferred 3D vision
10	Tubelo et al 2016, Brazil 40	Virtual learning object (VLO)	(46) 1st year DS	RCT	Theoretical knowledge and skill practice of zinc phosphate cement	Zinc phosphate cement manipulation after immediate or longitudinal access to VLO	VLO showed significantly higher results in theoretical post-tests and better mechanical properties
11	Shahriari-Rad et al 2017, UK 37	hapTEL virtual dental workstation	(140) 1st year DS	CCT	Objective structured clinical examination (OSCE) and clinical skills examination (CSE)	Students’ psychomotor skills and spatial perceptions	Significant improvement in psychomotor skills. Combined use of hapTEL and conventional phantom-head improved spatial reasoning, fine motor skills, hand-eye-finger coordination and 3D/depth perception
12	Cox et al 2017, UK 38	hapTEL workstations	(138) 1st year DS	RCT	Students’ fine motor-skills	Hand-eye-finger movements (pre-, post-) % of caries removed, pulp exposure, and time Micro-CT scanning of excavated plastic teeth	Significant correlation between the pre- and post-test results, and time with caries removal % and negatively with pulp exposure. Roughness of the preparations varied amongst students
13	Al-Saud et al 2017, UK 12	Simodont VR haptic dental simulator	(63) Participants with no previous dental experience	RCT	Preparation of geometric shapes with device feedback, or instructor feedback or both (IDFB)	Acceptable target removal percentage of all tasks was 70%	Significant differences between groups in overall performance, with IDFB group substantially better in performance and fewer errors
14	de Boer et al 2017, Netherlands 23	Simodont dental trainer	(101) 1st year DS	CCO	Geometric cross preparation with or without force feedback (FFB) Questionnaire	Success if 90% of the red target area removed	Only students with FFB were able to pass the tests. 100% of the students preferred working with FFB
15	Gottlieb et al 2017, United States 41	VR Advanced simulation	(282) DS of three sequential dental classes	CT	Class I and II amalgam preparations and restoration, and Class III and IV composite restoration	Advanced simulation exams scores in operative dentistry and fixed prosthodontics	Advanced simulation exam scores 1 and 2 were predictors of performance in the two preclinical courses based on final course grades
16	Ria et al 2018, UK 24	hapTEL system	(39) 1st year DS	CST	Cavity preparation and caries removal of increasing difficulty	% of tissue removed, pulp exposure, time	Insignificant better performance with the hapTEL system, despite lower scores reported with increased difficulty
17	Mirghani et al 2018, UK 26	Simodont system	(289) Dental students	CCS	Six manual dexterity exercises, to remove a target “red zone”	% score of task completion Drill time (in seconds)	Significant difference in performance between year 1 and years 4 and 5. Year 3 was significantly different to year 5
18	Dwisaptarini et al 2018, Thailand 25	Visuo-tactile virtual reality simulator connected to two haptic devices	(32) 6th year DS	RCT	Pre- and post-training clinical assessment of carries removal on extracted tooth	Performance scores Tooth mass loss and task completion time	Post-training performance significantly improved for both groups with insignificant differences between groups
19	Llena el al. 2018, Spain 13	AR cavity models on computers and mobile devices	(43) 3rd year DS	RCT	Theoretical knowledge before, immediately and 6 mo after training Clinical skills Satisfaction questionnaire	10 theoretical concepts Class I and Class II cavity preparation Students’ satisfaction	Insignificant differences in knowledge between groups but significant in cavity depth and extent for Class I and Class II cavities. Students preferred computers over mobile devices
20	de Boer et al 2019, Netherlands 27	Simodont haptic dental trainer	(126) 1st year DS	CST	Successful drilling with alternating FFB Post assessment questionnaire	A preparation on one block cross-figure Participants’ perception of the study	83% of the students passed the test. Skill transfer from one level of FFB to another was feasible with sufficient training
21	Vincent et al 2020, France 28	haptic simulator (Virteasy)	(88) 1st year DS	RCT	Both groups took final exam on plastic analogue teeth	Cavity preparation	Improvement in the drilling skill of both groups with insignificant differences
22	Murbay et al 2020, Hong Kong 29	VR–based system (Moog Simodont)	(32) 2nd year DS	RCT	Cavity preparation evaluation based on SISTA classification	Prepared cavity depth and width, and marginal ridge integrity	Satisfactory domains were significantly higher in experimental group and no significant difference between the manual and digital methods of evaluation
23	Osnes et al 2021, UK 30	Simodent, HT for removal of carries	(111) 1st year DS and 17 clinical practitioners	CST	Removal of virtual carious lesion spreading along the amelodentinal junction (ADJ)	Precision score	Clinicians were significantly more precise than students in removing caries without excessively removing the noncarious areas
Abbreviations: AR, augmented reality; CCO, comparative crossover; CCT, case control trial; CS, cohort study; CST, cross sectional trial; DS, dental students; FFB, force feedback; RCT, randomized controlled trial; VR, virtual reality.							

#### Endodontics

Six of the selected studies applied VR in endodontic with total included participants, *n* = 189. Characteristics of the selected studies are shown in Table 2 . HT was applied for access opening in three studies, 43 44 45 and surgical apicectomy in two studies. 14 15 VR simulation was used in one study to teach root canal anatomy. 46 Four studies showed significant better results of the virtual technology. 14 43 44 46 Students highly appreciated virtual training in one study, 15 although suggested modifications in spatial registration precision, FFB of different tissues, and more realistic models in another study. 45

**Table 2:** Characteristics of the selected studies in endodontics

S. no	Author, Year, Country	VR system	Participants	Study design	Assessment tool	Tested outcome	Results
1	Pohlenz et al 2010, Germany 15	Medified Voxel-Mann virtual simulator with haptic feedback	(53) DS of different years	CST	Students performed virtual apicectomies and responded to a questionnaire	1–5 scale to detect simulator is useful, realistic, sufficient, and desirable	The students indicated that FFB, spatial 3D perception, and image resolution of the simulator were sufficient
2	Suebnukarn et al 2010, Thailand 43	VR haptic simulator with augmented kinematic feedback	(32) 4th year DS	RCT	Virtual access cavity preparation in upper 1st molar (Three groups received kinematic augmented feedback and one control group did not)	Performance scores	The three kinematic feedback groups significantly scored higher with no significant difference in between
3	Suebnukarn et al 2011, Thailand 44	VR haptic simulator	(32) 4th year DS	RCT	Access cavity preparation was assessed before and after training for both groups on an extracted tooth	Procedural errors assessed by an expert	Post training error scores improved significantly for both groups. Hard tissue loss was significantly less in the haptic group, but not time
4	Suebnukarn et al 2012, Thailand 14	VR haptic simulator	(10) Post graduate endodontic trainees	RCT	Endodontic microsurgery of apicectomy	Endodontic competency scale by two experts	Significant higher scores of trials performed after virtual presurgical training
5	Wang et al 2015, China 45	iDental surgical simulator with a haptic device	(10) Fresh-graduate DS, (10) Residents	CST	Two dental drilling tasks:1-carries removal, 2- pulp chamber opening Subjective evaluation questionnaire	Time and amount of tissue removed	Insignificant differences between groups, though the residents spent more time. Dentists’ showed positive attitudes toward the system
6	Reymus et al 2020, Germany 46	VR environment	(32) 3rd year DS	CST	Root canal anatomy studies on periapical radiographs, CBCT scan and virtual reality environment	Post training knowledge questionnaire	CBCT or VR had significant better results than periapical radiograph. Most students’ preferred method of studying dental anatomy was VR
Abbreviations: CBCT, cone beam computerized tomography; CST, cross sectional trial; DS, dental students; FFB, force feedback; RCT, randomized controlled trial; VR, virtual reality.							

#### Oral and Maxillofacial Surgery

Nine of the selected studies applied VR technologies in oral and maxillofacial surgery education with total included participants, *n* = 730. Characteristics of the selected studies are shown in Table 3 . Virtual patient (VP) simulation was applied in four studies, 47 48 49 50 AR in three studies, 51 52 53 and IVR in two studies. 54 55 Results showed significant differences in all the selected studies except one study. 53 Participants positively appreciated the value of the VR in education, and the test groups reported significantly higher self-confidence.

**Table 3:** Characteristics of the selected studies in maxillofacial surgery and oral pain

S. no	Author, Year, Country	VR system	Participants	Study design	Tool of assessment	Tested outcome	Results
1	Clark et al 2012, United States 47	Autonomous virtual patient (AVP)	(26) 4th year DS, (10) board experts	CT	Examination of four VP with orofacial pain or oral medicine problem	Examination time, number of diagnostic tests, number of medications	Significant differences in the final total score, the number of diagnostic tests ordered, and the number of medications selected
2	Pulijala et al 2018, India 54	IVR surgery to train Le Fort-1 surgery	(95) Surgical residents	RCT	1. Pre- and post-training self-assessment of perceived confidence 2. Objective cognitive skills assessment	1. Self-confidence 2. Change in knowledge of surgical residents	Study group showed significantly greater perceived self-confidence but insignificant differences in knowledge scores
3	Seifert et al 2019, Germany 48	VP on e-learning platform “Lernbar”	(57) 4th year DS	RCT	Theoretical tests; pre, immediately after T1, and 6-wk T2 Self-assessment questionnaire	MCQs for structured facial examination and placing a venous catheter and Ernst ligature Self-assessment of knowledge and competency	VP group scored better than control group at T1 and no difference at T2. Both interventions led to a significant growth in self-assessed competence
4	Mladenovic et al 2019, Serbia 51	AR simulator on mobiles	(41) 4th and 5th year DS	RCT	Application of local anesthesia Post-clinical knowledge questionnaire	Knowledge and skills. Measurement of heartbeat during anesthesia administration	The experimental group had higher average score, less time of administration, and higher success rate. Both groups had a statistically significant increase in heart rate
5	Mardani et al 2020, Iran 49	Web-based VP in clinical decision-making ability	(76) DS	Quasi experiment	Knowledge pre-, post- (1 wk), and post-training (1 mo) Questionnaire on procedural knowledge	Procedural knowledge Problem-solving ability	Clinical decision-making score of VP group was significant more than the control group in post-test 1 but control group scores rose significantly more in post-test 2
7	Mladenovic et al 2020, Serbia 52	Mobile AR simulator	(11) 4th year DS	CST	Simulated local anesthesia (infiltrations and nerve block) then electronic satisfaction survey	Student satisfaction	All respondents (100%) believe (agree and strongly agree) that the application helped them to better understand the techniques of local anesthesia
6	Sakowitz et al 2020, United States 53	VP of complex orthognathic cases	(30) 3rd year DS	RCT	Knowledge pre- (T0), post- (T1), and follow-up test (T2) Written case analysis of two cases	MCQs score Case analysis score	No significant difference between the groups in MCQs examinations and the written case analysis
8	Collaço et al 2020, Brazil 55	IVR in inferior alveolar nerve block anesthesia	(163) DS	CT	Technical skills Participants’ subjective experience with syringe handling and simulator sickness	Task execution time, insertion accuracy, insertion point coordinates, needle angle, and needle depth	IVRs were significantly more accurate and confident and took less time. No significant differences in needle angle and needle depth. Participants perceived a high sense of realism with the haptic feedback when handling the syringe
9	McAlpin et al 2020, United States 50	Web-based patient simulator (Web-Sim)	(221) DS	RCT	Cognitive, psychomotor, and professional interpersonal skills in local anesthesia and nonsurgical extraction	Student-recorded role-paly video MCQs	Web-Sim group scored significantly higher in the role-play videos but insignificant MCQs scores
Abbreviations: AR, augmented reality; CT, comparative trial; CST, cross sectional trial; IVR, immersive virtual reality; MCQs, multiple choice questions; RCT, randomized controlled trial; VP, virtual patients.							

#### Prosthodontics

Thirteen of the selected studies applied VR in prosthodontics with total included participants, *n* = 815. Characteristics of the selected studies are shown in Table 4 .

**Table 4:** Characteristics of the selected studies in prosthodontics

S. no	Author, Year, Country	Technology	Participants	Study design	Assessment tool	Tested outcome	Results
1	Kikuchi et al 2013, Japan 58	DentSim, VR simulation (VRS)	(43) 5th year DS	RCT	Porcelain fused to metal crown preparation	Total scores included 12 preparation items and time	VRS scores were significantly higher. Instructor’s feedback did not result in significant difference within VRS groups
2	Hamil et al 2014, United States 59	Surface mapping technology E4D for students’ grading	(81) DS	CST	Students’ perception questionnaire	Students’ attitudes on the effectiveness of software in developing clinical skills	Students preferred digital grading over traditional and found the software helping them to understand their deficiencies
3	Eve et al 2014, United States 60	3D immersive haptic simulator	(12) novice DS, (12) experienced prosthodontics residents	CT	Simulated caries removal exercise	Percentages of carious lesion removed, and volume of surrounding sound tooth structure removed	Efficiency of carries removal improved significantly for both novice and experienced subjects
4	Callan et al 2014, United States 61	E4D Laboratory works virtual simulation using CAD/CAM technology	(76) 2nd year DS	RCT	CES within the intervention group (1st effectiveness analysis) and between the two groups (2nd efficacy analysis)	Full gold crown preparation on tooth #30. Students’ scores before and after using E4D and using E4D versus not. Post training and post-exam survey	1st effectiveness analysis showed no difference in outcomes. 2nd efficacy analysis showed insignificant higher mean competency scores of CAD/CAM group. Students appreciated the subjectivity of system’s evaluation and the beneficiary in tooth surfaces reduction
5	Lin et al 2018, United States 56	3D instructional models’ application on smartphones	(90) 2nd year DS	CST	Instruction models on rest seat preparation then a questionnaire	Evaluate students’ usage and perceptions of the digital models	73% of the participants who viewed the models responded either agree or strongly agree to the benefits of the models
6	Liu et al 2018, China 62	Online Peer-Review System (OPRS) and Real-time Dental Training and Evaluation (RDTES)	(66) 4th year DS	RCT	Post-training preparation of an anterior ceramic crown on phantom model Questionnaires	Pre-defined 15 evaluation criteria of the ceramic crown preparation Students’ attitude	Digital group was significantly better than the traditional group and 96.97% of it agreed or strongly agreed on the clinical benefits of the system
7	Kozarovska and Larsson 2018, Sweden 63	Digital tool for preparation Validation (PVT)	(57) 3rd year DS	CCO	All-ceramic crown in anterior teeth “prep. and scan” or “best of three” Students’ questionnaire and teachers’ opinions	The level of agreement between the students’ self-assessment and the information from the PVT	“prep-and- scan” showed increase in agreement from attempt one to three, with PVT. In “best of three” lower levels of agreement. Students rated PVT positively and teachers’ feedback suggested improvement modifications
8	Nagy et al 2018, Hungary 64	Dental Teacher software	(36) 4th year DS	RCT	Ceramic mesio-occluso-buccal on lay in a plastic model, scanned and assessed by Dental Teacher software	Six cavity evaluation parameters	Three of the six cavity dimension parameters improved significantly in the test group
9	Liu et al 2020, China 65	Virtual Real-time dental training and evaluation System (RDTES)	(57) 5th year DS	CST	Ceramic crown preparation, pre- and post-learning assessment Questionnaire	Instrument selection, preparation section, reduction, surface and profile	Mean total outcome score after VR training was significantly higher except in mean error score. 97% agreed or strongly agreed that the virtual system could improve their practice
10	Tang et al 2021, China 66	Digital real-time evaluation system (DCARER)	(60) DS, (73) Prosthodontic residents, (10) faculty members	RCT	Crown preparation process and final scores Questionnaire	Agreement between DCARER scores and expert Comparison between groups’ crown preparation scores	Insignificant differences between DCARER and experts’ scoring Tooth preparation scores of the traditional group were significantly lower. More students in the digital group believed the judgment of DCARER is more objective
11	Serrano et al 2020, Netherlands 3	HT models of real patients added in Simodont	(10) 4th and 5th year DS	CST	Training on real patient-haptic volumetric models, then in real patient Final open answer survey	Perceived learning value of the technology and self-assessed confidence and limitations	Identifiable five dimensions of the main features of VR: added value, competence development, self-efficacy, outcomes, and room for development
12	Mai et al 2020, Korea 57	3D simulated graphic dental models and computer designed Software	(60) 2nd year DS	RCT	After the course, 1. An attitudinal survey 2. Final examination	Assessing the preference of participants Knowledge test on the principles of adjustment of deflective occlusion	Students’ feedback indicated that the 3D simulation method was effective in acquiring knowledge on occlusion. Examination scores were significantly higher in the 3D simulation group
13	Al-Saud et al 2020, UK 67	Simodont haptic simulator	(72) 4th year DS	RCS	Students’ scores at year 2 on traditional training or haptic VR training	Full crown test preparation on patient in year 4	VR haptic simulator assessment score was a significant predictor of clinical crown performance
Abbreviations: CES, competency exam scores; CCO, comparative crossover; CT, comparative trial; CST, cross sectional trial; DS, dental students; RCS, retrospective cohort study; RCT, randomized controlled trial; VR, virtual reality.							

All studies applied VR in fixed prosthodontics training and evaluation, except two studies: one in preclinical removable partial denture prosthodontics course, 56 and the second in teaching occlusion. 57 Manual skills of tooth preparation was evaluated in nine of the selected studies, 58 59 60 61 62 63 64 65 66 67 acquired knowledge in one study, 57 and students’ perception in three studies. 3 56 59 Nine studies reported significant statistical differences of the VR scores. 57 58 60 61 62 63 64 65 66 67

#### Implantology

Five of the selected studies applied dental implant education with total included participants, *n* = 351. Characteristics of the selected studies are shown in Table 5 . Implant placement manual skills were assessed in four studies, 68 69 70 71 and theoretical knowledge in two studies. 70 72 Results of all the selected studies showed significant improvement of implant education outcomes in both clinical skills and theoretical knowledge.

**Table 5:** Characteristics of the selected studies in implantology

S. no	Author, Year, Country	VR system	Participants	Study design	Assessment tool	Tested outcome	Results
1	Qi et al 2013 China 72	Active and passive controlling 3D virtual webpages	(95) 1st and 2nd year DS	RCT	Post-training assessment of knowledge on dental implant restoration	Relative quality of information acquisition	Passive 3D control had significant high scores, a significant correlation existed between the scores on a mental rotations test and the subjects’ performance on the post-test
2	Joseph et al 2014, France 68	Virteasy, haptic dental simulator (implant surgery)	(40) 3rd year DS, (20) Experienced practitioners	RCT	Implant drilling in the 1st molar region in a custom-made mandibular resin model	Accuracy of implant placement and drilling times	The results of the simulator group were significantly close to the experienced operators
3	Golob Deeb et al 2019, United States 69	Dynamic guidance system software for virtual implant placement	(14) Predoctoral students	CST	Five implant placements (3 maxillary or 4 mandibular) positions	Surgical time horizontal, vertical, and angulation discrepancies	Significant reduction in time from 1st to 2nd trial, then plateaued. 3D angulation and 2D vertical apex deviation improved with each attempt, but changes in lateral 2D and overall 3D apex deviations were not significant
4	Zhang et al 2020, China 70	VR simulation platform	(166) 2 ^nd^ and 3 ^rd^ year DS	RCT	Pre- and post-theoretical test, subjective evaluation of operation procedures, implant accuracy in CBCT, and questionnaire	Procedural accuracy vs. jaw-bone simulation Degree of satisfaction	VR combined with jawbone groups had significantly higher increase in scores and showed better implant precision in CBCT than the other groups. Students preferred the combined of jawbone and VR reality simulation
5	Zorzal et al 2021, Brazil 71	IMMPLANT VR simulator uses smartphone and laptops	(16) dental postgraduates	CST	Place a virtual implant at a specific bone-loss area location within a subject-specific 3D model of a lower jaw	Participants feedback regarding benefits and limitations	VR system is easy to use and promotes greater spatial awareness of the 3D dental model and easy to learn but they reported difficulty selecting the predetermined implant position and inclination
Abbreviations: CST, cross sectional trial; DS, dental students; RCT, randomized controlled trial; VR, virtual reality.							

#### Oral and Maxillofacial Radiology

Two studies reported the application of VR in dental radiology education with total included participants, *n* = 84. Characteristics of the selected studies are shown in Table 6 . Both studies reported significant improvement of students’ skill to interpret spatial information in radiographs and acquisition of theoretical knowledge, although OSCE scores were insignificantly different. 73 74

**Table 6:** Characteristics of the selected studies in oral and maxillofacial radiology

S. no	Author, Year, Country	VR system	Participants	Study design	Assessment tool	Tested outcome	Results
1	Nilsson et al 2011, Sweden 73	VR simulator-supported training	(45) 4th and 5th year DS	RCT	Comparison of base line and after intervention theoretical examination	Skill at interpreting spatial information in radiographs	Radiographic interpretation skills 8 mo after simulator-supported training was significantly better than before training
2	Soltanimehr et al 2019, Iran 74	Virtual learning management system (LMS)	(39) 4th year DS	RCT	Theoretical test with MCQs and objective structured clinical examination (OSCE) at base line and after 2 mo	Radiographic interpretation of bony lesions	Scores of the virtual group were significantly higher in theoretical exam but insignificant in OSCE. After 2 mo difference was not statistically significant
Abbreviations: DS, dental students; MCQs, multiple choice questions; OSCE, objective structured clinical examination; RCT, randomized controlled trial; VR, virtual reality.							

#### Periodontology

Two studies considered HT in periodontology with total included participants, *n* = 55. Characteristics of the selected studies are shown in Table 7 . HT features were evaluated as high realistic in periodontal tasks, 75 and significantly improved pocket probing scores. 76

**Table 7:** Characteristics of the selected studies in periodontology

S. no	Author, Year, Country	VR system	Participants	Study design	Assessment tool	Tested outcome	Results
1	Wang et al 2012, China 75	iDental haptic-based simulator	(19) Dental graduates, (10) faculty members	CST	Virtual tasks of periodontal pocket probing, and calculus detection and removal, followed by user questionnaire	Realism of the simulator relative to clinical situations	Participants reported highly realistic shape of teeth, gingivae, periodontal tools, and oral environment, but poor realistic shape of the calculus and FFB
2	Yamaguchi et al 2013, Japan 76	Haptic-based simulator	(26) 4th year DS	CST	Carries removal and periodontal pocket probing in three training sessions	Carries removal. Periodontal pocket probing skills	The mean scores from the training sessions were significantly higher than the mean pre-training score for both carries removal and periodontal pocket probing skills
Abbreviations: CST, cross sectional trial; DS, dental students; FFB, force feedback; VR, virtual reality.							

#### Pediatric Dentistry

Four studies applied VR in pediatric dentistry with total included participants, *n* = 295. Characteristics of the selected studies are shown in Table 8 . Pediatric VP significantly improved behavior and communication management, 77 and AR significantly improved infiltrative anesthesia administration time. 78 Students highly perceived HT in the training on pediatric clinical tasks, 79 and VR superimposing 3D holograms in local anesthesia administration. 80

**Table 8:** Characteristics of the selected studies in pediatric dentistry

S. no	Author, Year, Country	VR system	Participants	Study design	Assessment tool	Tested outcome	Results
1	Papadopoulos et al 2013 in Greece 77	VR simulation pediatric VP	(103) 4th year DS	RCT	MCQs knowledge questionnaire VP feedback	Students’ knowledge of behavior and communication pediatric dentistry	VP group had significantly higher scores and the majority evaluated the aspects of the simulation very positively
2	Mladenovic et al 2020 in Serbia 78	AR simulator	(21) Fourth and fifth year DS	RCT	The time taken to administer the anesthesia. Level of salivary cortisol before and after Level of salivary cortisol before and after the administration of anesthesia	Perception of learning and acute stress level	AR group reported significantly shorter time. The level of cortisol significantly increased no statistical difference between the groups
3	Zafar et al 2020, Australia 79	Simodont Haptic simulator	(100) Doctorate degree students	CCO	Traditional and simulator training on pulpotomies and stainless-steel crowns (SSCs), followed by a questionnaire	Experience of pulpotomy and SSCs procedures on the Simodont, vs. conventional training	Over 50% agreed that Simodont-assisted learning, and facilitated understanding of pediatric dentistry tasks, although they felt more comfortable with the conventional training setup
4	Zafar et al 2021, Australia 80	Oculus Quest (VR headset plus digital 3D holograms and 360-degree spatial sound)	(71) Second year DS	CST	Self-administered questionnaire before and after the use of dental LAVR simulator	Dental student’s perception of dental LAVR simulation on a pediatric patient	Most of the participants reported improved LA skills, more engaged in the learning activity, improved understanding of anatomical landmarks, and added value compared with traditional LA teaching methods
Abbreviations: AR, augmented reality; CCO, comparative crossover; CST, cross sectional trial; DS, dental students; LAVR, local anesthesia virtual reality; MCQs, multiple choice questions; RCT, randomized controlled trial; VP, virtual patient; VR, virtual reality.							

#### Orthodontics

One study considered VR in orthodontics education. The study applied Scenario Based Learning Interactive software (SBLi) on orthodontics postgraduates, *n* = 9. Participants reported a high acceptance rate of the package, greater confidence applying the clinical skills covered in the modules, and reduced contact time. 81

#### Miscellaneous Dental Skills

Eight studies applied virtual strategies in teaching miscellaneous dental skills; critical thinking, 82 professionalism, 83 scientific writing, 84 knowledge of home dental practice, 85 head and neck anatomy, 86 dental morphology, 87 dental diagnosis, 88 and social aspects of dental care delivery. 89 Total included participants were *n* = 543. Characteristics of the selected studies are shown in Table 9 .

**Table 9:** Characteristics of the selected studies in miscellaneous dental skills

S. no	Author, Year, Country	VR system	Participants	Study design	Assessment tool	Tested outcome	Results
1	Allaire 2015, United States 82	VP in critical thinking assessment	(31) Senior hygiene DS	CST	Pre- and post-theoretical MCQs test and questionnaire	Skills of critical thinking, problem solving, and confidence	Insignificant increase in students’ scores although they reported VP an effective teaching method in enhancing self-confidence with real patients
2	Marei et al 2018, Saudi Arabia 83	Five VP for teaching professionalism	(65) First year DS	CST	Structured questionnaire before and after training	Students’ perception toward the use of VPs in developing ethical reasoning skills	High-fidelity VPs were significantly better for developing ethical reasoning skills
3	El Tantawi et al 2018, Saudi Arabia 84	DentLit video game to develop academic writing skills	(92) First year DS	Quasi experiment	Pre- and post-intervention assessment of students’ academic writing skills	1. Satisfaction of students with gamification 2. Perceived and actual improvement of academic writing	Significant improvement in actual writing. Overall satisfaction with game aspects was modest and significantly associated with improvement of writing
4	Takagi et al 2019, Japan 85	IVR for teaching home dental practice	(101) DS	CST	Survey before and after watching the VR teaching material	Changes in self-confidence regarding knowledge of home dental practice and treatment assistance	A significant increase in student’s knowledge confidence and assistance confidence scores
5	Zafar and Zachar 2020, Australia 86	HoloHuman AR to teach head and neck anatomy	(88) Second year DS	CST	Self-administered questionnaire before and after the use of AR	Perceptions of the AR	AR improved anatomical structures learning and understanding, and they felt more confident, but it should not replace traditional cadaver training
6	Liebermann and Erdelt. 2020, Germany 87	VR in learning dental morphologies	(48) Second year DS	CST	Questionnaire	Students’ acceptance	Most of the students understood dental morphologies much better compared with traditional textbook
7	Tsai et al 2020, United States 88	Mobile multimedia platform to teach dental diagnosis	(89) Predoctoral DS	CST	Baseline and 4-d later theoretical test and questionnaire	Basic dental diagnostic skills	Test scores increased significantly. Most students agreed on the ease of access and use of the platform and preferred Instagram stories over traditional lectures
8	Amini et al 2021, United States 89	IVR to teach social aspects of dental care delivery	(29) Dental residents	CST	Pre, immediately after and after 1-mo survey	Knowledge, skills, and attitude toward social determinants of health	Significant increased mean scores for cognitive, affective, and skill-based learning immediately post-training and no significant changes after 1-mo. Participants reported high satisfaction with the content and methods used in this training
Abbreviations: AR, augmented reality; CST, cross sectional trial; DS, dental students; IVR, immersive virtual reality; VP, virtual patients; VR, virtual reality.							

## Discussion

The application of VR in dental education has evolved increasingly, and there is significant scientific evidence that describes different virtual setups in different dental educational modules. However, the actual significance of VR simulation on dental education outcomes is not entirely clear. Earlier, VR may have been considered luxurious or optional, nevertheless in the shadow of the global COVID-19 (coronavirus disease 2019) pandemic, dental students need to proceed with their curriculum without any setbacks of the physical presence. VR may provide an opportunity for dental students to build and retain theoretical and clinical dental expertise remotely.

This systematic review showed that VR significantly enhanced the acquisition of dental manual skills even in short periods of training and, to a lesser extent, retention of theoretical knowledge. Despite the fact that few studies reported longer periods of follow-up and reported insignificant differences between virtual and traditional groups. 39 48 49 74

The diversity in students’ learning styles and motivation is the crucial challenge which course designers face. The introduction of virtual simulators in the dental curriculum and the utilization of its data to stratify dental students and predict their clinical performance would provide the opportunity to tailor the learning process to meet individual diversity in students’ expertise and allow students to work at their own pace. In this context, the dental curriculum could provide an education that leads to the optimal performance of each student. 26

Based on the results of this review, five broad, interrelated areas of significance arose; first, the versatility of VR applications and the increased application in some dental disciplines over others; second, HT and its wide use in dental education; third, the development of virtual dental patients to enhance dental education; fourth, the value of digital real-time feedback; and fifth, the access of students to the virtual technology.

First, VR applied in dental education showed a wide range of devices and applied technologies ranging from VR simulation with or without immersive environment, haptic simulators with or without force feedback, AR devices, real-time digital mapping and evaluation, virtual mobile platforms, video games, and other forms of virtual packages. The diversity of the individualized detailed features reflects the fact that there are no well-known educational standards for dental simulators or associated exercises. Additionally, it is doubtful how the variable reliability of the simulator systems may affect dental education outcomes. 6 Taking into consideration the complexity of the required dental training to reach a high degree of clinical competence, most of the studies included in this review applied VR in restorative dentistry, prosthodontics, and oral and maxillofacial surgery. In contrast, few studies represented pediatric dentistry, dental radiology, periodontology, and orthodontics. Restorative dental tasks might offer the feasibility of customization of the required assignments, whereas other dental disciplines may require higher customization and knowledge to fulfill specific field’s requirements. 90

Second, this review showed that HT was the most used technology, especially in tasks that require drilling and tooth preparations, which agree with Towers et al. 6 HT offers an additional dimension to VR through the sense of touch and force feedback (FFB) of the different tooth-layered structure and bone. Thus, HT proved efficient in training junior dental students the hand-eye coordination and spatial reasoning skills. It also helped students improve the preparation accuracy, shortened the preparation time in the very early stages of training, and augmented a conservative preparation approach. 15 22 37 68 However, due to the unique character of dental procedures, FFB should be improved and included as an integral feature in any educational dental simulator to enhance the perception of the tooth structure and different layers of bone. Training with FFB provides a sense of realism and allows the learner to obtain the feel of an invasive procedure in a virtual learning environment. 23 27

Third, VP showed wide applications in dental education and had a significant positive impact on manual skills and theoretical knowledge acquisition. VP reduced anxiety associated with real patient’s management while executing a treatment plan, exposed students to an interactive learning experience, enriched self-assessed competence, and augmented confidence to deal with actual patients. As simulators offer flexibility in terms of time, this allowed the students to repeat the procedure until they demonstrate acceptable skill levels without violating real patients and eliminating the need for prolonged direct contact. 47 48 49 53 77 Still, VP for dental training requires further development to simulate the patient’s oral environment of gingival tissues, saliva, tongue movements, and reflexes as gagging, cough, and head movements. Accordingly, it would aid in teaching emergency management in the dental setting. 75

Fourth, VR applications with real-time dental training and evaluation systems were very beneficial in acquiring motor skills in preclinical settings. It allowed instantaneous feedback of the students’ performance, enhanced students’ self-assessment, and correction and eliminated the subjectivity of evaluation. 59 64 65 Nevertheless, dental students indicated that the simulating devices’ instructions and feedback should be adjunctive to but not a replacement to the faculty feedback. Faculty should be attentive to their responsibility in teaching young dentists, treating patients with individual needs, requiring empathy and informed consent for any treatment decision. The faculty’s role-model function is essential when supervising students during patient treatment in clinical practices, complex problem solving, in-depth conceptual coverage, and peer interaction. Continuous training with faculty supervision and feedback is still an anticipated key to good dental education.

Fifth, most of the studies applied VR through academic laboratories, a fact that should be reconsidered, and alternative mobile platforms should be developed. To benefit from the technology, the student must be physically present on the academic campus. This situation limits to a great extent the range of getting most of the benefit of the virtual technology due to the condensed academic timetables and the increased training times required. Meanwhile, curriculum designers should notice that virtual applications on personal computers and mobiles might leave the whole education process in the student’s hands, for whom some can organize their time accordingly, while others cannot. Thus, supervisors and teachers must monitor the learning process since a lack of motivation in some students would downgrade the technology’s benefit. 13 In this context, tutors should operate continuous assessment in the form of pop-up quizzes, group discussions, and scheduled assignments or presentations, which would eventually lead to a blended form of learning, highlighting the teacher’s role. 48

Based on the results of this review, it is recommended that low-cost VR hard and software be made readily available to create safe and cost-effective interactive educational training, allowing learners and trainees instantaneous engagement through their personal computers or mobiles. It is advised to clarify learning contents and the extent to which conventional workflows should be taught, aside from the virtual content. One form of a teaching strategy that should be utilized on a wider scale is educational video games. This form of educational material elevated students’ enthusiasm for learning and made learning an enjoyable process. 42 84 Young generations are more prominent in adapting to new technologies and increasingly familiarized with video games, encouraging further development and improvements in this field to introduce education with more fun.

## Limitations

Our study has several limitations. The retrospective nature of our review, incorporating data from published studies and not on individual patients, limits the availability of information on some issues as long-term follow-up of the students and the influence of VR on clinical practices. The search process revealed heterogenous studies addressing the systematic review’s aim, and while meta-analysis was not feasible, we conducted a descriptive approach for identifying the effective outcome of virtual applications. Custom-made software was only used by authors who first described them, which is a significant flaw and could represent a conflict of interest in validating a new proposed system. Also, there was a lack of randomized clinical trials with a proper sample size calculation and other efforts to avoid major bias.

## Conclusion

Advanced simulation technology improved the quality of dental education outcomes. It offered applications in different dental disciplines and various clinical procedures. HT enhanced manual skills and perceived self-confidence within few clinical sessions. The most remarkable improvement was the cavity walls convergence, pulpal floor, extension of class I, cavity outline, fewer pulpal exposure, and faster preparation. Students performed better in 3D than 2D vision, with FFB than without, and with a combined instructor and device feedback than with instructor or device feedback alone. Quality of crown preparation and implant placement improved over time after using VR with or without instructor’s feedback. AR reinforced orthognathic surgical training, virtual apicectomies, and local anesthesia administration. Application of VR improved acquisition of theoretical knowledge to a lesser extent. The role of the teacher and verbal instructions cannot be ruled out.
